# AXDND1 is required to balance spermatogonial commitment and for sperm tail formation in mice and humans

**DOI:** 10.1038/s41419-024-06874-5

**Published:** 2024-07-12

**Authors:** Brendan J. Houston, Joseph Nguyen, D. Jo Merriner, Anne E. O’Connor, Alexandra M. Lopes, Liina Nagirnaja, Corinna Friedrich, Sabine Kliesch, Frank Tüttelmann, Kenneth I. Aston, Donald F. Conrad, Robin M. Hobbs, Jessica E. M. Dunleavy, Moira K. O’Bryan

**Affiliations:** 1 School of BioSciences and Bio21 Molecular Sciences and Biotechnology Institute, The University of Melbourne, Parkville, VIC Australia; 2 Instituto de Investigação e Inovação em Saúde, Universidade do Porto, Porto, Portugal; 3 Centro de Genética Preditiva e Preventiva, Instituto de Biologia Molecular e Celular, Universidade do Porto, Porto, Portugal; 4 Division of Genetics, Oregon National Primate Research Center, Beaverton, OR USA; 5 Genetics of Male Infertility Initiative (GEMINI) Consortium, Beaverton, OR USA; 6 Centre of Medical Genetics, Institute of Reproductive Genetics, University of Münster, Münster, Germany; 7 Centre of Reproductive Medicine and Andrology, University Hospital Münster, University of Münster, Münster, Germany; 8 International Male Infertility Genomics Consortium (IMIGC), Newcastle-upon-Tyne, UK; 9 Department of Surgery (Urology), University of Utah, Salt Lake City, UT USA; 10 Centre for Reproductive Health, Hudson Institute of Medical Research, Clayton, VIC Australia; 11 Department of Molecular and Translational Sciences, School of Clinical Sciences, Monash University, Clayton, VIC Australia

**Keywords:** Infertility, Microtubules

## Abstract

Dynein complexes are large, multi-unit assemblies involved in many biological processes via their critical roles in protein transport and axoneme motility. Using next-generation sequencing of infertile men presenting with low or no sperm in their ejaculates, we identified damaging variants in the dynein-related gene *AXDND1*. We thus hypothesised that AXDND1 is a critical regulator of male fertility. To test this hypothesis, we produced a knockout mouse model. *Axdnd1*^*−/−*^ males were sterile at all ages but presented with an evolving testis phenotype wherein they could undergo one round of histologically replete spermatogenesis followed by a rapid depletion of the seminiferous epithelium. Marker experiments identified a role for AXDND1 in maintaining the balance between differentiation-committed and self-renewing spermatogonial populations, resulting in disproportionate production of differentiating cells in the absence of AXDND1 and increased sperm production during initial spermatogenic waves. Moreover, long-term spermatogonial maintenance in the *Axdnd1* knockout was compromised, ultimately leading to catastrophic germ cell loss, destruction of blood–testis barrier integrity and immune cell infiltration. In addition, sperm produced during the first wave of spermatogenesis were immotile due to abnormal axoneme structure, including the presence of ectopic vesicles and abnormalities in outer dense fibres and microtubule doublet structures. Sperm output was additionally compromised by a severe spermiation defect and abnormal sperm individualisation. Collectively these data identify AXDND1 as an atypical dynein complex-related protein with a role in protein/vesicle transport of relevance to spermatogonial function and sperm tail formation in mice and humans. This study underscores the importance of studying the consequences of gene loss-of-function on both the establishment and maintenance of male fertility.

## Introduction

Spermatogenesis is the process wherein sperm are produced and involves three broad processes: (1) stem cell (spermatogonia) renewal and proliferation via mitosis to produce spermatocytes, (2) the process of meiosis in spermatocytes where chromosome number is halved, and (3) the complete transformation of round haploid germ cells into spermatozoa with the potential for motility via processes collectively known as spermiogenesis. All aspects of spermatogenesis are dependent on microtubule-mediated protein and organelle transport [1, 2]. This is particularly evident during the process of spermiogenesis wherein the sperm assembly is achieved [3]. During spermiogenesis multiple related transport pathways exist, including along cytoplasmic microtubules through classical protein transport pathways, the bi-directional transport of proteins along the microtubules of the manchette via a process called intra-manchette transport (IMT) and along the microtubules of the axoneme via the classical, in addition to likely modified versions of the intra-flagellar transport (IFT) pathway [4, 5]. A family of complexes essential for many of these processes is the dyneins [6, 7]. There are two types of dynein complexes, the cytoplasmic dyneins, which transport cellular cargoes along microtubules towards their minus end within the cell cytoplasm [8–11], and axonemal dyneins, which hydrolyse ATP to drive the beating of all motile cilia, including the sperm tail [12–14].

The canonical cytoplasmic dynein complex is composed of a dimer of two dynein heavy chains to which two intermediate chain, two light intermediate chain and three light chain dynein subunits bind [8, 15]. In contrast to cytoplasmic dyneins, axonemal dynein complexes are made up of three heavy chain subunits (α-, β- and γ-), alongside a unique set of light chain subunits not previously associated with the cytoplasmic dynein complex [16]. In both classes of dynein complexes, the heavy chain dynein components act as the motor/engine of the complex. The heavy chains each possess a microtubule-binding domain adjacent to an AAA module [16, 17], which facilitates ATP hydrolysis to power complex movement along microtubules or cell motility. Following assembly of the core dynein complex, other regulatory proteins are recruited to enhance activity and specify which cargoes (passengers [cytoplasmic]) or adaptors can bind to each complex sub-type [18, 19]. Different adaptor (auxiliary) protein combinations exist within different tissues and are likely involved in different biological processes [20]. The full suite set of functions dynein complexes take part in spermatogenesis are yet to be fully elucidated [4].

Previously we used whole exome sequencing on a group of infertile men with azoospermia (no sperm in their ejaculate) and identified a novel biallelic stop-gain variant in the dynein-related gene *AXDND1* [21]. While AXDND1 contains a dynein light chain domain and is thus annotated as an axonemal dynein light chain-containing protein, it is notably different from the typical light chain dyneins. Specifically, at 120-125 kDa, AXDND1 is considerably larger than bona fide mammalian dynein light chain proteins (~8–30 kDa), and does not contain other domains typical of light chain dyneins [9]. AXDND1 does however contain a conserved PF10211 domain, homologous to the core dynein domain in DNALI1 and *Chlamydomonas* p28 [9, 22]. While untested, we predict this domain likely confers AXDND1 the ability to interact with dynein heavy chains, the large motor protein subunits that drive cell motility or protein transport complex motility along microtubules [8]. Previously it has been shown that the loss of *Axdnd1* function in mice results in male infertility due to its role in sperm head shaping via the manchette and tail assembly during spermiogenesis [23, 24]. This phenotype was, however, a poor match with the azoospermic clinical presentation of the infertile man we identified to be carrying a stop-gain variant.

To further test the role of AXDND1 in male fertility, we generated an *Axdnd1* knockout mouse line and rigorously assessed the effects of AXDND1 loss on germ cell development and male fertility. We aimed to test if *AXDND1* was a human male infertility gene and validate its link to azoospermia in the infertile men containing *AXDND1* variants. We also aimed to investigate the spermatogenic processes AXDND1 is involved in, using histological and sperm functional assays. Specifically, we investigated the role of AXDND1 in germ cell commitment to spermatogenesis during early postnatal development (days 3–7) and the role of AXDND1 in testis germ cell maintenance, spermatid remodelling and sperm function over a range of 40–180 days of age.

## Materials and methods

### Mouse model production

The structure for human *AXDND1* and mouse *Axdnd1* transcript information (ENSG00000162779 and ENSMUSG00000026601, respectively) are shown in Supplementary Fig. 1 and data was sourced from Ensembl. To test the requirement for *AXDND1* in male fertility, *Axdnd1* knockout (*Axdnd1*^*−/−*^*)* mice were generated by the Monash Genome Modification Platform (MGMP; Monash University, Australia) using CRISPR/Cas9 technology on C57BL/6J mice. Excision of exon 5 (ENSMUSE00001068050) of the longest *Axdnd1* transcript ENSMUST00000213088.1 (Supplementary Fig. 1; transcript 211) was achieved using CRISPR guide sequences shown in Supplementary Table 1 and led to a premature stop codon in exon 6. The deletion was confirmed and characterised by Sanger sequencing. This approach generated two founder lines: one with a 564 bp deletion and 5 bp insertion (*Axdnd1*^*del1*^ line) and another with a 571 bp deletion (*Axdnd1*^*del2*^ line) – both contained a premature stop codon in exon 6. After mating founder mice to wild-type C57BL/6J mice, resulting mutant *Axdnd1*^*+/−*^ mice were used to generate *Axdnd1*^−/−^ individuals. *Axdnd1* expression levels for the longest transcript (*Axdnd1-*211) and a smaller transcript (*Axdnd1-*208) that does not contain exon 5 (and thus was not targeted by the CRISPR deletion) were assessed in testis samples via qPCR as previously described [25], using primers shown in Supplementary Table 1.

### Analysis of *Axdnd1* expression

Whole organ RNA was extracted from adult mouse brain, epididymis, heart, liver, lung, spleen and testis to investigate *Axdnd1* expression across tissues. RNA was also extracted from the testes of mice aged 0–50 days to investigate *Axdnd1* expression throughout the first wave of spermatogenesis. Primers used to detect *Axdnd1* and housekeeping gene *Ppia* are shown in Supplementary Table 1. As a direct measure of *AXDND1* and *Axdnd1* germ cell expression, we also utilised single-cell RNA sequencing data generated through previous studies in our lab [26, 27].

### Fertility analysis of *Axdnd1*^−/−^ males

Fertility analyses were performed on males from the *Axdnd1*^*del1*^ line over an age range of 30–180 days. Phenotyping was also performed on adult males from the *Axdnd1*^*del2*^ line at 70–90 days of age. The fertility and reproductive parameters of wildtype and *Axdnd1*^−/−^ males were assessed using the pipeline outlined in [28]. In brief, at least four mice of each genotype were assessed for fertility at two age points (42–50 and 70–80 days) by mating with two young wild-type females each. These ages equate to just after the first wave of spermatogenesis and epididymal maturation has completed, and mature adult males, respectively. Animals were not allocated or randomised into groups – rather they were assessed based on genotype. Sample size was estimated based on severity of the phenotype and experience in each assay. Investigators were blinded to genotype prior to analyses.

Sperm motility was assessed via computer-assisted sperm analysis as described previously [29], including in an additional experiment where 1 mM of membrane permeable ATP (ATP disodium salt hydrate [A2383 – Merck, Australia]) was added in an attempt to rescue motility. Remaining sperm (and immature germ cells) were washed in 1 × PBS then fixed in 4% paraformaldehyde for 10 min and resuspended in PBS. Cells were then settled on SuperFrost+ slides overnight at 4 °C for further analysis. In additional experiments, epididymides were collected for calculation of epididymal sperm content [30], and testes and epididymides were collected for and fixed for transmission electron microscopy analysis as detailed below.

### Histological assessment of reproductive tissues and sperm/epididymal luminal cell content

Testis sections were stained with periodic acid-Schiff’s and haematoxylin reagents (PAS), while epididymis sections were stained with haematoxylin and eosin. Sperm and other cells settled to SuperFrost+ slides were stained with haematoxylin and eosin. All slides mounted in DPX under a 1.0 × coverslip for light microscope analysis using an Olympus BX-53 microscope fitted with an Olympus 392 DP80 camera.

To quantify the age-related changes in testis histology, the incidence Sertoli cell-only tubules, severe hypospermatogenesis and vacuoles, were counted at 40, 70 and 180 days of age, *n* = 3 mice/age/genotype. Sertoli cell-only was assigned when histology showed no germ cells, while severe hypospermatogenesis (approaching Sertoli cell-only) was recorded when very few germ cells were present. Intact tubules contained no defects in each of the three categories. Round spermatid numbers were counted at postnatal day 22 in a minimum of 10 stage V equivalent tubule cross-sections (round and oval tubules) per animal to investigate germ cell commitment to spermatogenesis (*n* = 5/genotype).

To quantify apoptosis in the testis of 40-, 70-, and 180-day-old males, testis sections were co-stained with 0.5 μg/ml cleaved caspase 3 (Cell Signalling Technology, Australia, Ab 9664) and 0.5 μg/ml cleaved caspase 7 (Cell Signalling Technology, Australia, Ab 9491) antibodies. Caspase-positive cells were counted in at least 50 tubules chosen randomly per male (*n* > 3 mice/genotype/age).

To investigate immune cell infiltration, testes were immersed in Tissue-Tek O.C.T. reagent (Sakura Finetek, CA, USA) prior to freezing in an ethanol dry ice slurry. Frozen testes were cryo-sectioned onto SuperFrost+ slides then stained with CD45 (BD Pharmingen [BD Biosciences] 550539) and 0.5 µg/ml smooth muscle actin primary antibody (Sigma A2547) antibodies overnight at 4 °C, after which they were stained with appropriate secondary antibodies, followed by 10 µg/ml DAPI (Thermo Fisher Scientific).

AXDND1 localisation was investigated in germ cells and sperm via tubule squash preparations as per [31]. Fixed germ cells were incubated in 6 µg/ml AXDND1 antibody (Sigma HPA071114) overnight at 4 °C and stained with appropriate secondary antibodies, 2 µg/ml wheat germ agglutinin-Alexa Fluor 555 (acrosome marker; Thermo Fisher Scientific W32464) and 10 µg/ml DAPI. Localisation of AXDND1 via immunohistochemistry and the use of western blotting on whole testis lysate failed to produce specific staining or bands.

#### Investigation of the blood–testis barrier integrity and immune infiltration

A biotin tracer (EZ-Link™ NHS-Biotin; Thermo Fisher Scientific, Australia) was used to test the integrity of the blood–testis barrier in *Axdnd1*^−/−^ mice as described previously [32]. Sections were stained with 0.5 µg/ml smooth muscle actin primary antibody (Sigma A2547) overnight at 4 °C, followed 1 µg/ml donkey anti-mouse secondary and 2 µg/ml streptavidin-Alexafluor-488 reagent (to visualise biotin, Thermo Fisher Scientific). Nuclei were counterstained with 10 µg/ml DAPI (Thermo Fisher Scientific) and slides mounted with Dako fluorescence mounting medium (Agilent).

#### Investigation of spermatogonia dynamics during the first wave of spermatogenesis

To quantify spermatogonia numbers, including those committing to spermatogenesis, during the first wave of spermatogenesis, we investigated germ cell content in testis sections at postnatal days 3–7 and 22–35. Testis sections were stained with the germ cell marker MVH (day 3–7; 3 µg/ml, Abcam ab13840), the undifferentiated spermatogonia markers PLZF (day 5–35; 0.6 µg/ml, R&D Systems AF2944) and FOXO1 (day 7; 1 µg/ml, Cell Signaling Technology #2880), and differentiating spermatogonia marker c-KIT (day 7; 0.6 µg/ml, R&D Systems AF2944) overnight at 4 °C. Sections were co-stained with 0.5 µg/ml smooth muscle actin antibody (Sigma A2547) and incubated with relevant secondary antibodies, then 10 µg/ml DAPI to stain DNA. The number of tubules with positive staining for MVH or PLZF, as well as the germ cells positive for each marker (MVH, PLZF, FOXO1 and c-KIT), in at least 20 round tubules was counted (*n* ≥ 3 animals/genotype). Round spermatid numbers were also counted in at least ten tubules of PAS-stained sections at postnatal day 22 (*n* = 5 animals/genotype).

#### Electron microscopy

To investigate germ cell and sperm ultrastructure, testes were fixed, processed and imaged as per [30] To view the sperm tail ultrastructure, sperm were isolated from the cauda epididymis and incubated in 100 µl of 1 × PBS for 30 min to strip the plasma membrane as previously described [33].

### Statistical analysis

Statistical analyses to determine significance between wild-type and *Axdnd1*^−/−^ parameters at each age point were performed in Prism (GraphPad, San Diego, USA) using an unpaired *t*-test with *α* = 0.05. Where data were not normally distributed, a non-parametric test was used (Mann-Whitney U). For age series data, a t-test was used to compare genotypes at each age point. Significance of apoptosis data was assessed as previously described [34].

### Study approval

#### Patient description

As described previously [21], we used exome sequencing on infertile men presenting with azoospermia or severe oligozoospermia to investigate novel genetic causes of male infertility. Herein, we identified additional variants in *AXDND1*. Variants in patients 2 (M1557) and 3 (M2628) were identified in the Male Reproductive Genomics (MERGE) cohort including 2412 participants (*n* = 2127 men with severe/extreme oligo-, crypto- or azoospermia) according to [35]. Testicular biopsies for histological examination are described in [36]. Experimental ethics for patients 2 and 3 were approved by the Ethics Committee of the Ärztekammer Westfalen-Lippe and the Medical Faculty Münster (ethics number 2010-578-f). Informed consent was obtained from all subjects.

#### Animal ethics

Experimental procedures involving mice followed animal ethics guidelines generated by the Australian National Health and Medical Research Council (NHMRC). All animal experiments were approved by the Animal Experimentation Ethics Committee (BSCI/2017/31) at Monash University, Clayton or The University of Melbourne Animal Ethics Committee (application 20640).

## Results

### A loss of function variant in AXDND1 is associated with azoospermia and human male infertility

**Fig. 1:**
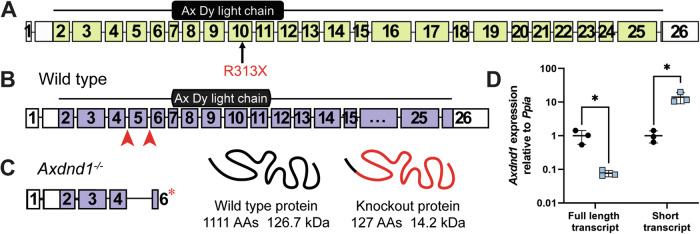
The *AXDND1* genetic variant and *Axdnd1*^−/−^ mouse model. **A** We identified a high-confidence disease-causing p.R313X stop-gain variant (red) in *AXDND1* in an infertile man with azoospermia. Human AXDND1 is encoded by 26 exons, with the stop-gain variant affecting exon 10, within the predicted axonemal dynein light chain domain. White exons are untranslated regions, green shaded exons are protein-coding. **B** Full-length mouse AXDND1 protein is encoded across 25 exons, with the dynein light chain domain encoded in exons 7-11. Red arrows denote guide RNA sites for generation of the knockout model via CRISPR. Purple-shaded boxes represent protein-coding exons, while the white portion of exon 25 is the 3′ untranslated region. **C** AXDND1 protein length in *Axdnd1*^*−/−*^ mice, comprising of exon 2–4 and a truncated exon 6 with a premature stop codon. Wild-type AXDND1 is represented in a basic form at 1111 amino acids in length and encoding 126 kDa of protein, while the red portion of the knockout protein represents the premature stop codon and the resulting 1064 amino acids that are not translated. **D** Expression of the full-length *Axdnd1* transcript and a smaller transcript that is not affected by the genetic modification were measured by qPCR, normalised to *Ppia* expression (*n* = 3), *p < 0.05. Data are presented as mean ± SD.

As part of the GEMINI study to define genetic causes of male infertility, we undertook exome sequencing of infertile men presenting with azoospermia or severe oligozoospermia and identified an azoospermic Portuguese man (patient 1) carrying a homozygous stop-gain variant in the dynein gene *AXDND1* (c.937T>C; p.R313X) (Fig. 1A) (first reported in [21]). This variant affected exon 10 of 26 in *AXDND1*, a region that encodes a predicted axonemal dynein light chain (PF10211) domain. As identified by the variant effect predictor in gnomAD, the p.R313X variant is high confidence for a loss of AXDND1 function. The CADD score for this variant is 42, which places it in the top 0.01% of deleterious variants in the genome. The predicted effect of this stop-gain mutation is nonsense-mediated decay of all the full-length RNA transcripts (*AXDND1*-201, 202 and 214; Supplementary Fig. 1), resulting in a loss of *AXDND1* expression, i.e., it is equivalent to a full loss of function mutant in a mouse.

A comparison of the *AXDND1/Axdnd1* protein-coding transcripts between human and mouse (Supplementary Fig. 1) revealed that three transcripts in human (*AXDND1-*201, -202 and -214) and four in mouse (*Axdnd1-*202, -204, -208 and -211) contained the PF10211 axonemal dynein light chain domain (exons 7–11).

### AXDND1 is enriched in haploid male germ cells

**Fig. 2:**
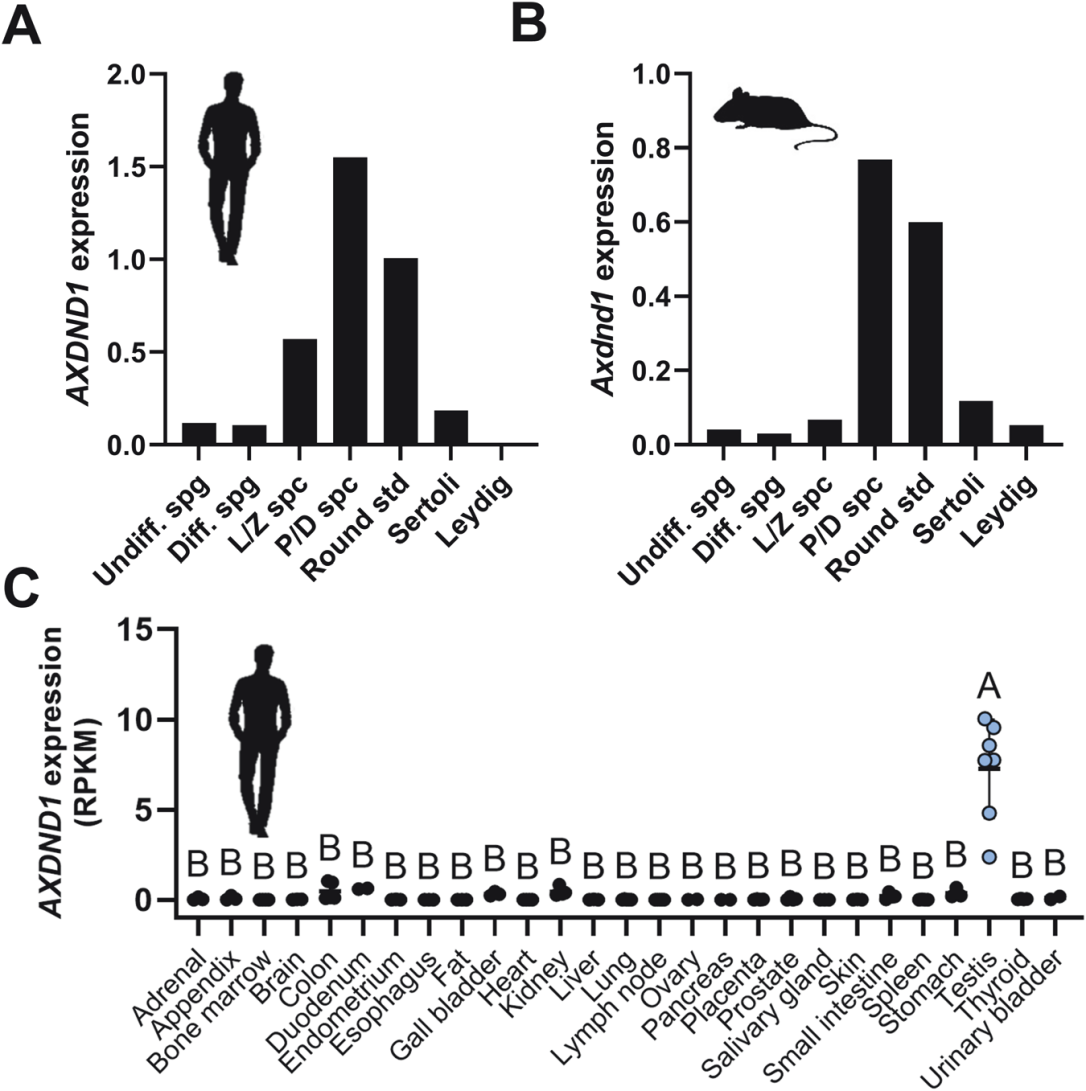
*AXDND1/Axdnd1* expression in testes and other organs. **A** Human *AXDND1* and **B** Mouse *Axdnd1* relative expression in testicular cells as identified by single-cell RNA sequencing. L-R: undifferentiated spermatogonia, differentiated spermatogonia, leptotene and zygotene spermatocytes, pachytene and diplotene spermatocytes, round spermatids, Sertoli cells and Leydig cells. **C** *Axdnd1* expression in human tissues (*n* = 2–7), as sourced from [54].

We investigated *AXDND1* expression using published testis single cell sequencing data [26, 27]. *AXDND1*/*Axdnd1* was expressed in all germ cell types, including spermatogonia, but most highly expressed within late spermatocytes and round spermatids in both men and mice (Fig. 2A, B). Assessment of *AXDND1* expression across several human tissues revealed a clear enrichment in testes (Fig. 2C; *p* < 0.0001).

### AXDND1 is essential for male germline maintenance and fertility in mice

To test the role of AXDND1 in male fertility, we generated a knockout mouse model to recapitulate the predicted complete loss of function of the p.R313X *AXDND1* variant identified in infertile patient 1 (Table 1). We used CRISPR to remove exon 3 of the principal *Axdnd1* transcript and introduce a premature stop codon in exon 4 (Fig. 1B, C). Two founder mouse lines were generated. Following the confirmation of comparable phenotypes in both lines, our analyses focused on the *Axdnd1*^*del1*^ line (described in the methods). Loss of expression of the full-length *Axdnd1* transcript in this knockout line was confirmed via qPCR (Fig. 1D), while the expression of a shorter transcript, encoded by exons 7–17 (*Axdnd1* transcript 208, Supplementary Fig. 1) was upregulated approximately tenfold (*p* < 0.05) compared to expression in wild-type testes. Transcript 208 encodes a truncated protein of 55 kDa (compared with 126 kDa for the full-length protein) that contains the predicted PF10211 domain.

**Table 1:** Variants identified in AXDND1 in infertile men with azoospermia or severe oligozoospermia.

Patient	Country of origin	Semen parameters and histology	*AXDND1* variant/ClinVar accession number			Pathogenicity
1	Portugal	Non-obstructive azoospermia, no biopsy	c.937C>T	p.Arg313Ter	Hom	LP
			VCV003236169.1			
2 M1557	Croatia	Non-obstructive azoospermia, Sertoli cell-only	c.1607T>A	p.Leu536Gln	Het	VUS
			SCV005038996			
			c.2602G>A	p.Ala868Thr	Het	VUS
			SCV005038997			
3 M2628	Turkey	Non-obstructive azoospermia, elongating spermatids in < 50% tubules	c.2451A>C	p.Lys817Asn	Het	VUS
			SCV005038998			
			c.2843A>G	p.Asp948Gly	Het	VUS
			SCV005038998			

*Hom* homozygous, *Het* heterozygous/phase unknown, *LP* likely pathogenic, *VUS* variant of unknown significance. ClinVar accession numbers are shown for each patient.

**Fig. 3:**
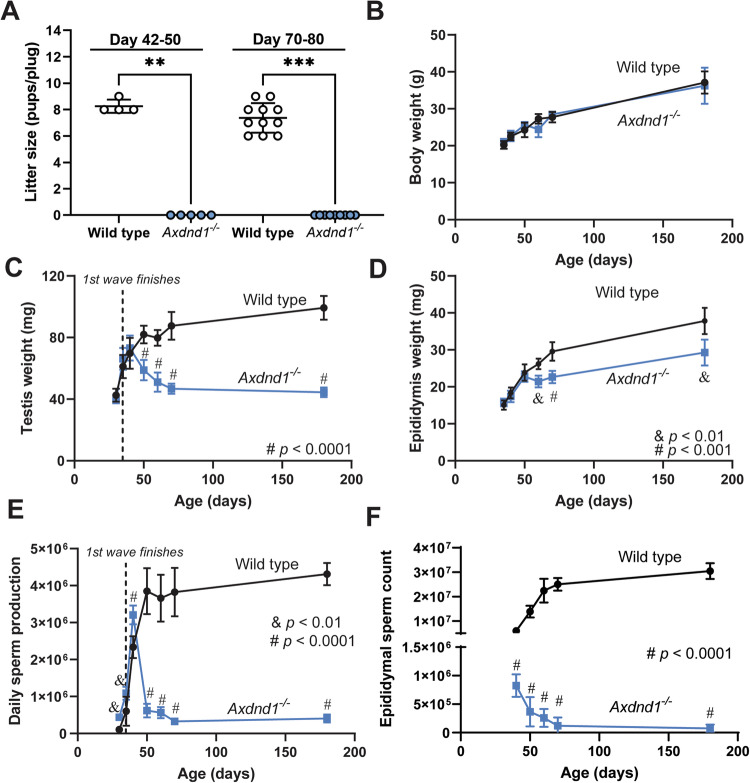
AXDND1 is essential for male fertility and normal sperm production capacity. Male reproductive parameters and body weight of wild-type and *Axdnd1*^−/−^ males were assessed across an age range of 35–180 days of age. **A** Male fertility as assessed by litter size for males mated just after the first wave of spermatogenesis (42–50 days of age) or after multiple waves. **B** Body weight. **C** Testis weight, including day 30 data points **D** Epididymis weight. **E** Daily sperm production, include day 30 data points. **F** Epididymal sperm count. ^&^*p* < 0.01; ^#^*p* < 0.0001 at respective age point. Data (all *n* ≥ 3 animals/time point/genotype) are presented as mean ± SD.

Initially, fertility of *Axdnd1*^*−/−*^ males was tested at two ages: (1) at the completion of the first wave of spermatogenesis and epididymal maturation (42-50 days of age) and (2) after several rounds of spermatogenesis (70–80 days of age). At both ages, *Axdnd1*^−/−^ males were sterile (Fig. 3A; *p* < 0.0001) although mating appeared normal as evidenced by the presence of copulatory plugs (0 pups/plug). Wild-type males plugged and sired several litters (average of 8.25 ± 0.5 and 7.36 ± 1.1 pups/plug, at postnatal days 42-50 and 70-80 respectively) at both ages. No differences were found in the body weight of *Axdnd1*^−/−^ males compared to wild-type across all ages investigated (Fig. 3B). Equally, seminal vesicle weights were unchanged between genotypes as a proxy for androgen levels (Fig. S2F).

Next, reproductive parameters of wild-type and *Axdnd1*^−/−^ males were assessed over an age range of 40-180 days (Fig. 3) to explore the role of AXDND1 in establishing the first wave of spermatogenesis and in the maintenance of spermatogenesis. Testis weights were comparable between wild-type and *Axdnd1*^−/−^ males until 40 days of age, after which a significant divergence was observed (Fig. 3C; *p* < 0.0001). *Axdnd1*^−/−^ testes weights progressively declined until 70 days of age, then remained constant through to 180 days of age. At postnatal day 180, *Axdnd1*^−/−^ testis weight was 45% of that measured from wild-type males. In accordance, epididymis weights were comparable between genotypes up until day 50 of age, then diverged from day 60 of age (Fig. 3D; *p* < 0.01). Such changes were suggestive of the successful completion of one round of spermatogenesis followed by age-related degeneration. To explore this possibility quantitatively, sperm production was measured as a function of age (Fig. 3E). During the first wave of spermatogenesis (30 and 35 days of age), knockout males generated significantly more sperm compared to wild type (*p* < 0.01). At 40 days of age, knockout males produced 30% more sperm than age-matched wild-type males (*p* < 0.0001). By 50 days of age, however, *Axdnd1*^−/−^ daily sperm production, decreased to 19% of wild-type levels (*p* < 0.0001). Similar levels of sperm production were maintained through to 180 days of age. Despite no initial increase in sperm number, an analogous pattern of reduced sperm number was observed within the epididymis as a function of age (Fig. 3F). At day 40 of age, sperm counts in *Axdnd1*^−/−^ epididymides were 12% of wild-type levels (*p* < 0.0001). Epididymal sperm counts then declined with age, and at 180 days of age were only 0.33% of wild-type levels. The disconnect in daily sperm production in the testis and epididymal sperm at day 40 was suggestive of a widespread spermiation defect. In support of this hypothesis, significant numbers of retained sperm were observed within stage IX–X tubules of day 40 *Axdnd1*^−/−^ testes (Supplementary Fig. 2B).

A comparable phenotype was observed in the second *Axdnd1*^−/−^ line (*Axdnd1*^*del2*^, Supplementary Fig. 3), thus confirming that the phenotype was specific to the loss of AXDND1 function and not an undetected ‘off-target’ CRISPR-mediated loss of gene function somewhere else in the genome.

**Fig. 4:**
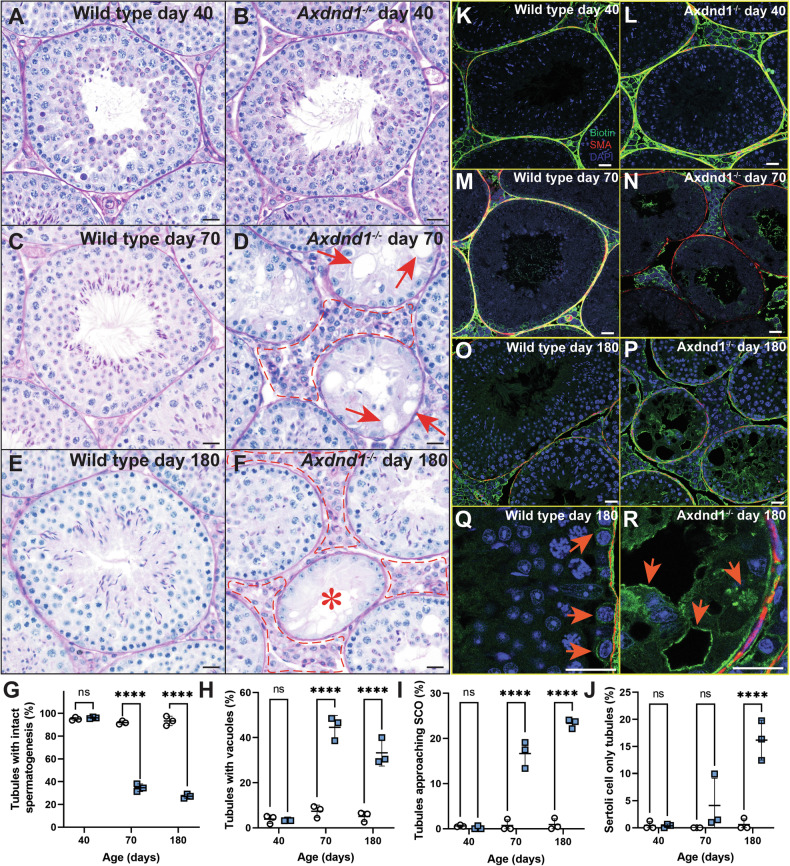
AXDND1 is required for normal and sustained spermatogenesis. Testis histology and apoptosis were assessed at days 40, 70 and 180 to investigate the role of AXDND1 in spermatogenesis. **A** Wild type and **B** *Axdnd1*^*−/−*^ knockout testes at day 40 of age. **C** Wild-type and **D** *Axdnd1*^*−/−*^ knockout testes at day 70 of age. Arrows point to vacuoles in the seminiferous epithelium. Red dashed lines denote abnormal interstitium. **E** Wild type and **F** *Axdnd1*^*−/−*^ knockout testes at day 180 of age. The asterisk denotes a Sertoli cell-only tubule, devoid of germ cells. Scale bars are 20 µm in length for all panels. **G** Assessment of seminiferous tubules with no abnormal presentation (intactness). **H** Assessment of tubules with vacuoles **I** Assessment of tubules with severe germ cell loss, approaching a Sertoli cell-only phenotype **J**. Assessment of tubules with a Sertoli cell only phenotype. **K** Assessment of apoptosis via caspase staining. **K**–**R** Wild type and *Axdnd1*^*−/−*^ testes were challenged with a biotin tracer and then fixed to investigate blood–testis barrier integrity. Biotin localisation (strepatividin-488 staining) is shown in green, smooth muscle actin (basement membrane marker) in red and DAPI in blue. Arrows in **Q** (wild type) denote an intact barrier, while arrows in **R** (*Axdnd1*^*−/−*^) denote biotin infiltration into tubule due to the loss of the barrier. Scale bars are 20 µm in length. *p < 0.05, ****p < 0.0001. Data (all *n* = 3) are presented as mean ± SD.

To explore the consequences of AXDND1 loss at a cellular level, we investigated testis histology (Fig. 4) in wild-type and *Axdnd1*^−/−^ males at 40 (immediately following the completion of the first wave of spermatogenesis), 70 (after several waves) and 180 (aged males) days of age. Testis histology was consistent in wild-type mice regardless of age Fig. 4). Analogous to the situation in wild-type mice (Fig. 4A), all adult germ cell types were present at 40 days of age in *Axdnd1*^−/−^ testes (Fig. 4B). By 70 days of age, however, and consistent with the changes in testis weight, spermatogenesis was overtly compromised as highlighted by a significant reduction in, or complete absence of, germ cells in a portion of seminiferous tubules, resulting in vacuoles in the seminiferous epithelium (Fig. 4D, red arrowheads). Associated with this was an influx of cells to the interstitial compartment of the testis (Fig. 4D, red dashed lines), which were confirmed to be immune cells by staining for CD45 (Supplementary Fig. 2D). These disruptions to spermatogenesis in knockout testes worsened by 180 days of age (Fig. 4F).

To quantify these changes in spermatogenic quality, the proportion of tubules presenting with complete spermatogenesis, hypospermatogenesis, or a Sertoli cell-only epithelium was quantified (Fig. 4G–J). These data revealed that the proportion of normal tubules with intact spermatogenesis decreased between 40 and 70 days of age in *Axdnd1*^*−/−*^ testes (Fig. 4G, *p* < 0.0001) and accordingly the proportion of tubules with vacuoles and tubules approaching Sertoli cell-only (severe hypospermatogenesis) in *Axdnd1*^−/−^ testes were both significantly elevated from 70 days of age (Fig. 4H, I; *p* < 0.0001). As a measure of complete germ cell loss, the number of complete Sertoli cell-only tubules was significantly elevated at 180 days of age (Fig. 4J, *p* < 0.0001). In agreement with this reduction in germ cell content, the quantitation of germ cell apoptosis via caspase staining revealed a significant increase in apoptotic germ cells in *Axdnd1*^*−/−*^ testes at 70 days of age compared to wild type (*p* = 0.02; Supplementary Fig. 2E).

As described in more detail below, those sperm present within the epididymis were immotile, thus explaining the origin of infertility in young adult males (Fig. 6). In addition to the severe tail defects, the loss of AXDND1 resulted in a significant increase in head shape abnormality (Supplementary Fig. 4). Objective sperm head shaping analysis revealed a reduction in the percentage of sperm heads classified as most normal (cluster 1, *p* = 0.0033) and an increase in the abnormal clustering (cluster 3, *p* = 0.023). This finding is in support of a critical role of AXDND1 in manchette dynamics [23, 24] and its localisation to the manchette.

### The blood–testis barrier is compromised in Axdnd1^−/−^ testes

The accumulation of immune cells within the interstitial space in older *Axdnd1*^*−/−*^ (Fig. 4D, F) males suggested the loss of integrity of the blood–testis barrier. The blood–testis barrier is a series of intercellular junctions between adjacent Sertoli cells that function to protect immunologically foreign meiotic and haploid germ cells from immune system attack [37]. To directly test the function of the blood-testis barrier we incubated testes with a biotin tracer (Fig. 4K–R). This approach revealed that the blood-testis barrier was intact in *Axdnd1*^−/−^ testes at 40 days of age as evidenced by the localisation of biotin exclusively within the interstitium and the basal compartment of the seminiferous epithelium which is external to the blood–testis barrier. By contrast, in 70- and 180-day-old *Axdnd1*^−/−^ testes the biotin tracer permeated deep within the tubules (Fig. 4N, P, R). As expected, the blood–testis barrier was intact in wild-type testes at all ages (Fig. 4M, O, Q). These data reveal that the loss of AXDND1 leads to a loss of blood–testis barrier integrity. It is unclear if this is a primary or secondary consequence of AXDND1 loss. Regardless, the loss of blood–testis barrier function would be expected to accelerate the seminiferous tubule degeneration with increasing age.

### AXDND1 is required for normal sperm tail development and function

**Fig. 5:**
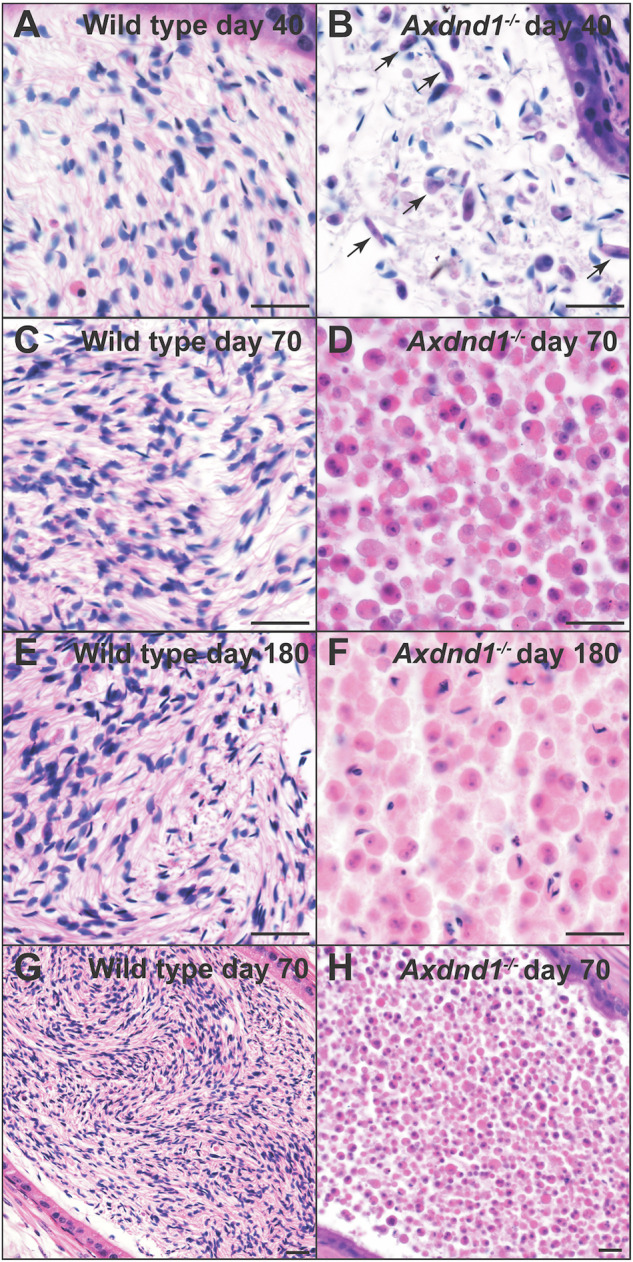
AXDND1 is required for normal sperm release into the epididymis. Cauda epididymis histology was assessed at days 40, 70 and 180 of age to investigate the cell types present. **A** Wild type and **B** *Axdnd1*^*−/−*^ knockout epididymides at day 40 of age. **C** Wild type and **D** *Axdnd1*^*−/−*^ knockout epididymides at day 70 of age. **E** Wild type and **F** *Axdnd1*^*−/−*^ knockout epididymides at day 180 of age. Lower power images of **G** Wild type and **H** *Axdnd1*^*−/−*^ knockout epididymides at day 70 of age are also shown. Scale bars are 10 µm in length.

The presence of sperm within day 40 *Axdnd1*^*−/−*^ testes and epididymides suggested the possibility of fertility in young animals. However, as described above, the mating experiments revealed males were sterile. To define the origin of this infertility, epididymis histology was examined at 40 days of age (Fig. 5). As outlined above, while sperm were present in the epididymis, numbers were decreased compared to age-matched wild-type males. By 70 days of age (Fig. 5D, H) very few sperm were seen and, instead, the cauda epididymis contained many round cells – likely prematurely sloughed germ cells as confirmed previously [24] and likely immune cells (Supplementary Fig. 2H). This finding was consistent in aged males at 180 days of age, where widespread phagocytosis of spermatid nuclei by immune cells was evident (Fig. 5F).

**Fig. 6:**
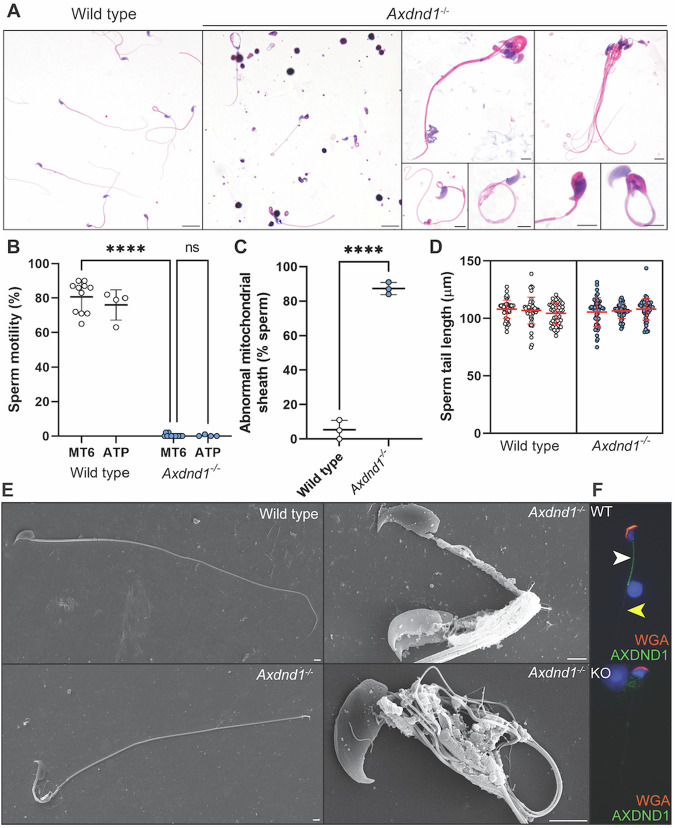
AXDND1 is an essential regulator of sperm tail development and required for sperm motility. **A** Sperm morphology of sperm from wild type (left panel) and *Axdnd1*^*−/−*^ (all other panels) stained with haematoxylin and eosin. Scale bars in large panels are 10 µm in length and in small panels are 5 µm in length. **B** Sperm motility in basal medium and with the presence of exogenous ATP. **C** Quantification of sperm mitochondrial sheath abnormalities. **D** Sperm tail length. **E** Scanning electron microscopy of sperm. Lower power panels (left) depict whole sperm external structure (plasma membrane removed). Higher power panels (right) focus on sperm midpiece / mitochondrial sheath regions in knockout. Scale bars are 2 µm in length. AXDND1 localisation in sperm collected via squash preparations (green), counterstained with DAPI (blue) and wheat germ agglutinin (acrosome marker, red). A white arrow denotes midpiece staining and a yellow arrow points to punctate principal piece staining. A negative control (sperm from knockout) is shown in the lower panel with no AXDND1 staining. **F** AXDND1 localisation in sperm collected via squash preparations (green), counterstained with DAPI (blue) and wheat germ agglutinin (acrosome marker, red). A white arrow denotes midpiece staining and a yellow arrow points to punctate principal piece staining. A negative control (sperm from knockout) is shown in the lower panel with no AXDND1 staining. Data (all *n* ≥ 3 animals/genotype) are presented as mean ± SD.

Of those sperm released by *Axdnd1*^−/−^ testes at day 40, the majority were abnormal (Fig. 5B, arrows), as highlighted by highly coiled sperm tails (Fig. 6A, E). Most possessed an abnormal midpiece region, abnormal head shaping and/or sperm that were not individualised correctly. Specifically, throughout most of spermatogenesis sister germ cells develop in a syncytium, wherein individual germ cells are separated by intracellular bridges [38]. During the final steps of spermatogenesis, residual cytoplasm is removed, and sperm are ‘individualised’ through largely unknown processes, but insights from flies reveal this process requires functioning actin and microtubule networks [39]. Sperm isolated from the cauda epididymis of *Axdnd1*^*−/−*^ males were completely immotile (Fig. 6B, *p* < 0.0001). Further, motility was not recovered with the addition of membrane-permeable ATP as an exogenous energy source, suggesting a core structural defect within the axoneme [4]. An analysis of midpiece/mitochondrial morphology revealed that the mitochondrial sheath of sperm was poorly formed or missing in 87% of sperm from *Axdnd1*^*−/−*^ males but only 5% of wild-type sperm (*p* < 0.0001; Fig. 6C). Sperm tail length was measured in individual sperm and revealed no significant differences between genotypes, noting this was not possible for those with coiled tails (Fig. 6D).

**Fig. 7:**
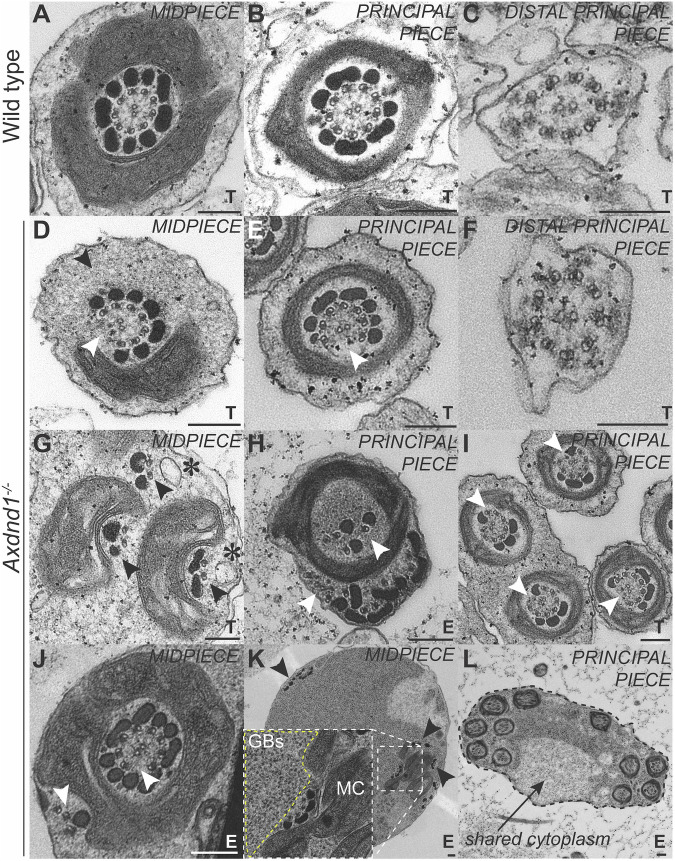
AXDND1 is a critical regulator of sperm axoneme ultrastructure. Transmission electron microscopy images of sperm axoneme cross-sections across all three regions of the sperm tail. Testis (T) versus epididymis (E) sperm samples are designated at the bottom right corner of each panel. **A**–**C**. Step 16 spermatids from wild type and **D**–**F** *Axdnd1*^*−/−*^ at the midpiece, principal piece and end piece level, respectively. The black arrow in D denotes missing mitochondria, and in D + E white arrows point to missing microtubule doublets and outer dense fibres (ODFs). **G**–**L**. Various defects in sperm tail ultrastructure in sperm from *Axdnd1*^*−/−*^. **G** Black arrows points to separate groups of microtubules and ODFs associated with mitochondria; asterisks denote vesicles associated with these microtubules. **H** White arrows point to disordered microtubules, including those displaced from the core exoneme encased within the fibrous sheath. **I**. White arrows point to missing microtubules and ODFs. **J** White arrows point to displaced microtubules. **K**. Black arrows point to disordered microtubules and ODFs throughout the cytoplasm. An insert shows granulated bodies (GBs, yellow dashed line) accumulated next to four microtubule doublets and ODFs associating with mitochondria (MC). **L** A black dashed line and arrow denotes a shared cytoplasm among several individual sperm tails. Scale bars are 200 nm in length.

A more detailed examination of cell ultrastructure via scanning electron microscopy on membrane-stripped sperm revealed that sperm generated by *Axdnd1*^*−/−*^ males had highly abnormal mitochondrial sheath structure and mitochondrial morphology (Fig. 6E). In contrast to the neatly coiled mitochondria of sperm from wild-type mice, those from *Axdnd1*^*−/−*^ males, possessed poorly coiled mitochondria and gaps within the sheath where no mitochondria were present. Further, in the midpiece compartment of sperm from *Axdnd1*^*−/−*^ males, microtubules and outer dense fibres splayed outward from the axoneme core and in some cases appeared to have snapped off. By contrast, the structure of the fibrous sheath of these sperm appeared to be largely normal as observed externally at a scanning electron microscopy level, but also internally as investigated by transmission electron micrographs (Fig. 7E, H, I).

To define why sperm from *Axdnd1*^*−/−*^ males were immotile, we used transmission electron microscopy on developing spermatids (testis) and released sperm (epididymal sperm) (Fig. 7). An investigation of step 15–16 spermatids, just before sperm release, and epididymal sperm from *Axdnd1*^−/−^ males revealed a battery of axoneme and other sperm tail defects (Fig. 7D–F) compared to those from developmentally matched cells from wild-type males (Fig. 7A–C). In most sperm, a portion of axoneme microtubule doublets were missing or ectopically positioned in both the midpiece and the principal piece. In distal principal piece sections, core axoneme ultrastructure, however, appeared broadly normal in sperm from *Axdnd1*^*−/−*^ males (Fig. 7F), suggesting defects were exacerbated during the loading of accessory structures. In addition, abnormal ODF loading was observed in virtually all cross-sections (Fig. 7D, E, G–J), as highlighted by absent or flimsy ODF structures. Further, we observed the over-accumulation of granulated bodies (precursor ODFs) in the cytoplasm (Fig. 7K inset – yellow dashed line; Fig. 7L). Ectopic vesicles were also identified in the axoneme (Fig. 7G, asterisks) and as outlined above, mitochondria loading in the midpiece was abnormal (Fig. 7D, G). All these defects are consistent with abnormal transport of proteins and organelles along microtubules – key functions of dynein complexes. Aligning with the finding of failed sperm individualisation, we detected many examples of multiple sperm sharing a common plasma membrane in samples from the epididymis (Fig. 7L).

To further explore if AXDND1 might play a direct role in organelle/protein transport within the sperm tail, we stained spermatids from wild-type and knockout males with an AXDND1 antibody. While the antibodies we tested did not result in specific staining in formaldehyde-fixed testis sections they did appear to be specific in elongating spermatids harvested using the dry-down method and fixed with paraformaldehyde [31]. As shown in Fig. 6F, AXDND1 localised strongly to the midpiece region of sperm and in a punctate manner in the principal piece in developing wild-type spermatids but not in knockout spermatids. AXDND1 also localised to the microtubules of the manchette and the developing head-tail coupling apparatus of spermatids (Supplementary Fig. 2F).

### AXDND1 is a regulator of spermatogonial commitment to spermatogenesis

**Fig. 8:**
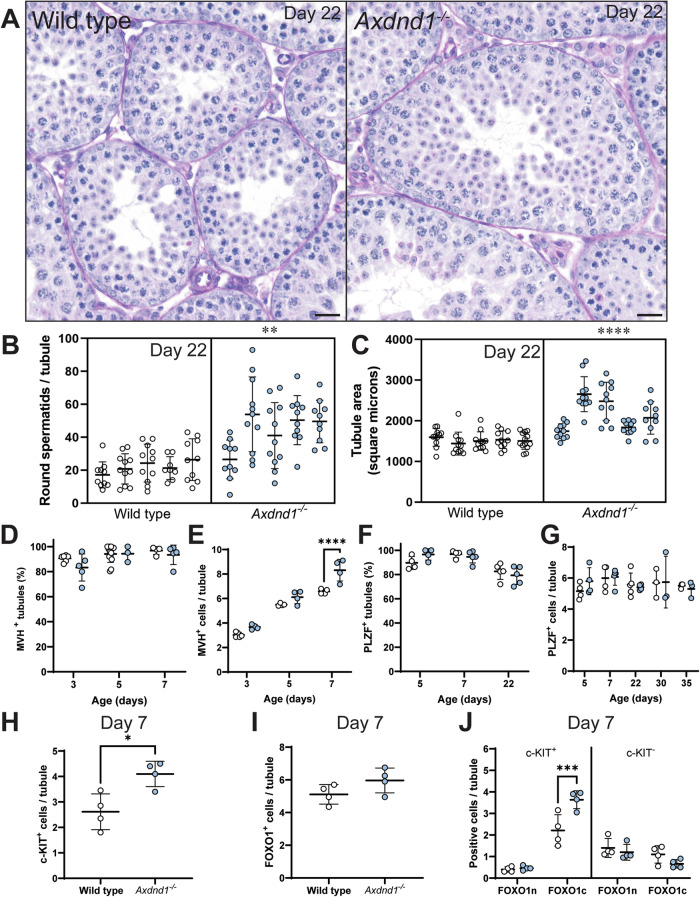
AXDND1 regulates spermatogonial commitment during the first wave of spermatogenesis. **A** Testis histology at postnatal day 22 of age, depicting larger tubules containing elevated round spermatid numbers in *Axdnd1*^*−/−*^ testes. **B** Round spermatid counts per tubule and **C** Tubule area at postnatal day 22. **D** MVH^+^ tubules and **E** MVH^+^ cell numbers per tubule at postnatal days 3, 5 and 7. **F** PLZF^+^ tubules and **G** PLZF ^+^ cell numbers per tubule at postnatal days 3, 5 and 7. **H** Numbers of c-KIT^+^ and **I** FOXO1^+^ cells per tubule cross-section at postnatal day 7. **J**. Numbers of FOXO1 nuclear (n) and cytoplasmic (c) cell numbers, separated into c-KIT^+^ (committed) and c-KIT^−^ spermatogonia populations at postnatal day 7. *p < 0.05, **p < 0.01, ***p < 0.001, ****p < 0.0001. Scale bars = 20 µm. Data (all *n* ≥ 3 animals/time point/genotype) are presented as mean ± SD.

The elevated daily sperm production measured in *Axdnd1*^−/−^ testes at days 30, 35 and 40 of age followed by a rapid decline is suggestive of AXDND1 playing a role in maintaining the overall balance between spermatogonial self-renewal and commitment to spermatogenesis. To explore this, we quantified the number of round spermatids at day 22 of age (Fig. 8A), where spermatids first appear at postnatal day 20 in wild-type males [40]. This analysis revealed a significant increase in round spermatid number in seminiferous tubules in *Axdnd1*^*−/−*^ testes compared to those from wild-type animals (Fig. 8B, *p* = 0.0026) and, accordingly, a significant increase in the average tubule area (Fig. 8C, *p* = 0.0071).

To define the origins of the increase in cell number, we quantified the numbers of MVH^+^ germ cells (pan germ cell marker) in testis sections at postnatal day 3–7 and PLZF^+^ cells (undifferentiated and early differentiating spermatogonia) from postnatal day 5, when nascent undifferentiated and differentiating spermatogonia have been generated from fetal germ cell precursors (known as gonocytes or pro-spermatogonia) [41], through to day 35 of age. Differentiating spermatogonia directly generated from gonocytes produce the first wave of spermatogenesis while subsequent sperm production is dependent on the undifferentiated pool that contains spermatogonial stem cells (SSCs). Importantly, the proportion of tubules containing MVH+ germ cells was unaffected in *Axdnd1*^−/−^ testes compared to wild-type controls during the first postnatal week, indicating that germline development occurred normally (Fig. 8D). Specifically, normal numbers of gonocytes populated the testis at postnatal day 3 in both genotypes, suggesting gonocyte proliferation was undisturbed. In wild-type testes, the number of MVH^+^ germ cells per tubule cross-section increased from postnatal day 3–7 as the nascent spermatogonial pool was established and started to divide (Fig. 8E). However, while the number of MVH^+^ cells per tubule cross-section in *Axdnd1*^−/−^ testes was unaffected at postnatal days 3 and 5 versus controls, it was significantly increased at day 7 (Fig. 8E, *p* < 0.0001), consistent with increased mitotic expansion and/or reduced apoptosis of germ cells [42, 43]. In contrast, the percentage of tubules containing PLZF^+^ spermatogonia and the number of PLZF^+^ cells per tubule cross-section were both comparable to controls at all ages assessed (Fig. 8F, G), suggesting that formation and initial maintenance of undifferentiated spermatogonia were unaffected by *Axdnd1* knockout.

To extend our analysis, we investigated staining for (1) c-KIT, a marker of differentiated spermatogonia, and (2) the transcription factor FOXO1, an independent marker of undifferentiated spermatogonia that is also expressed in gonocytes [44]. At postnatal day 7, numbers of c-KIT^+^ spermatogonia per tubule were significantly increased in *Axdnd1*^−/−^ testes compared to controls (Fig. 8H, *p* = 0.014), while the abundance of FOXO1^+^ cells was not significantly changed (Fig. 8I, *p* = 0.13). These data suggest that the observed increase in germ cells during postnatal testis development was due to increased numbers of differentiating rather than undifferentiated spermatogonia, consistent with normal numbers of PLZF^+^ spermatogonia in knockout testes (Fig. 8E–G). Importantly, subcellular localisation of FOXO1 is controlled dynamically during spermatogonial development and differentiation. FOXO1 is predominantly cytosolic in neonatal gonocytes, then translocates to the nucleus in undifferentiated spermatogonia but becomes cytosolic then downregulated upon spermatogonial differentiation [44, 45]. At postnatal day 7, numbers of c-KIT^-^ undifferentiated spermatogonia with predominantly nuclear FOXO1 were comparable between *Axdnd1*^−/−^ and controls (Fig. 8J). However, numbers of c-KIT^+^ spermatogonia with cytoplasmic FOXO1, i.e., spermatogonia at early differentiation stages, were significantly increased in the knockout at this timepoint (Fig. 8J, *p* < 0.001).

Combined, our data indicate that AXDND1 is not required for development of the foundational population of undifferentiated spermatogonia during postnatal development but that AXDND1 limits differentiation commitment from the precursor pool and/or survival of nascent differentiating spermatogonia [43], leading to increased sperm production during initial spermatogenic waves upon *Axdnd1* deletion. At later timepoints, germline integrity in *Axdnd1* knockout testis is lost, indicating that long-term maintenance of the undifferentiated population is dependent on AXDND1.

### Additional AXDND1 variants in infertile men

In order to search for additional men carrying *AXDND1* variants, cohorts of men within the International Male Infertility Genomics Consortium (imigc.org) were assessed. We identified four variants (Table 1): two nonsynonymous SNVs in a Croatian man (patient 2, M1557) with azoospermia and a Sertoli cell-only presentation, and two nonsynonymous SNVs in a Turkish man with azoospermia (patient 3, M2628), where a biopsy revealed less than 50% of seminiferous tubules bearing elongating spermatids (Supplementary Fig. 5). All four of these variants were classified as variants of unknown significance. While the phases could not be determined in both cases (segregation analysis and long read sequencing), the disease presentation is consistent with patient 1 wherein a complete loss of *AXDND1* function variant was identified. Collectively these data indicate that, analogous to the situation in the mouse, AXDND1 is required for the maintenance of human spermatogenesis.

## Discussion

Herein we reveal that AXDND1 is essential for male fertility for mice and men, functioning in several phases of spermatogenesis. AXDND1 plays a critical role in regulating the overall balance of spermatogonial commitment versus renewal during the first wave of spermatogenesis. AXDND1 is additionally required for the formation of functional sperm tail structures, sperm individualisation and head shaping, and spermiation. These latter functions are consistent with a role in regulating protein/organelle transport along microtubule structures. Exacerbating the consequences of AXDND1 loss, but highlighting the importance of cell-cell communication in spermatogenesis, while mice are still relatively young, the absence of AXDND1 leads to a loss of blood–testis barrier integrity and the infiltration of immune cells into the seminiferous epithelium. The latter will cause further damage to the seminiferous epithelium. While previous studies have identified AXDND1 to be essential for male fertility in mice, they have not identified the evolving phenotype of *Axdnd1*^*−/−*^ mice, the deficits in sperm individualisation, and the key role AXDND1 plays in spermatogonia and thus the longer-term integrity of spermatogenesis [23, 24]. Collectively, these data identify AXDND1 as an atypical dynein complex protein that plays multiple roles in mouse and human spermatogenesis.

Importantly, we demonstrated a previously unappreciated role for AXDND1 as a regulator of spermatogonial commitment to spermatogenesis. The increased numbers of differentiating spermatogonia in *Axdnd1*^−/−^ testes resulted in an elevated sperm number during the first wave of spermatogenesis. However, subsequent waves were disrupted due to progressive germline degeneration. How AXDND1 limits the production of differentiation-committed spermatogonia while ensuring long-term maintenance of undifferentiated states is unclear. This may be explained by it playing a role in the transport of specific cell-regulatory factors. Previous research has shown that the RNA-binding protein DAZL, which is essential for the translation of RNAs important for male germ cell development, interacts directly with the cytoplasmic dynein complex [46]. In addition, it was postulated that RNAs bound to DAZL could be transported by dyneins when required for developmental processes and protein translation [46]. AXDND1 may be involved in a similar process, acting as a dynein adaptor required for specific germ cell-regulatory factors.

AXDND1 was originally annotated as a light chain domain-containing protein of the axonemal dynein class. If this was the case, it would be expected that AXDND1 plays a role specifically in axoneme function to manifest sperm motility. We did observe a complete loss of motility in the absence of AXDND1. We, however, also identified several structural defects in sperm from *Axdnd1*^*−/−*^ males which suggest AXDND1 plays a more general role in protein or organelle transport. In support of a function in association with cytoplasmic dynein complexes, AXDND1 localised to the mitochondrial sheath and the neck of sperm, two structures that are involved in cytoplasmic mediated functions. By contrast, AXDND1 was localised only weakly to the principal piece, which forms solely within the ciliary compartment [4], providing only weak evidence that AXDND1 is a core component of the dynein arms of the axoneme.

AXDND1 also contains a PF10211 domain that is conserved in DNALI1, a dynein light intermediate chain which has been shown to interact with cytoplasmic dynein 1 [22]. A variant encoding hypomorphic DNALI1 in mice resulted in axoneme microtubule doublet and fibrous sheath abnormalities [47], suggesting this domain permits interaction with cytoplasmic dynein complexes. These data suggest AXDND1 may act as an adaptor to optimise the interaction between dynein complexes and cargoes (e.g., components of the ODFs) to allow their efficient transport along the microtubules of the manchette to the site of entry of the sperm tail compartment (cytoplasmic dynein 1) and/or via retrograde transport along the developing sperm tail (cytoplasmic dynein 2) in the ciliary compartment. The former interpretation is supported by the presence of abnormal concentrations of granulated bodies in the cytoplasm of elongating spermatids and a failure of mitochondrial loading in elongating spermatids from *Axdnd1*^*−/−*^ mice [48]. A role in cytoplasmic dynein complex 2 is supported by the presence of abnormal accumulations of vesicles in the axoneme and as highlighted previously [24]. AXDND1 does not, however, appear to play a role in core intra-flagellar transport that seeds the axoneme core of the sperm tail as evidenced by normal sperm tail lengths and a normal axoneme presentation in the end piece of sperm from the knockout model. *Axdnd1* null sperm tails were, however, immotile, and unresponsive to cell-permeable ATP, supporting that the transport and assembly of key components of the dynein arms into the microtubule doublets of the axoneme was compromised.

The loss of sperm individualisation has rarely been seen in mouse models. An additional light chain domain-containing dynein ‘ctp’ that encodes the same PF10211 domain, has, however, previously been shown to be essential for the process of sperm individualisation in flies [36], suggesting a potential conserved role for dyneins in this process. Recently, it was demonstrated that the process of sperm individualisation in mice is driven by an autophagy pathway [49] and likely requires efficient neddylation, a process of targeting cellular material for degradation that is similar to ubiquitination [50]. Whether AXDND1 aids in trafficking members of either pathway to sites for targeted removal of the cytoplasmic bridges and other material required for individualisation should be tested in future studies.

Herein where we describe AXDND1 localises to the manchette, and in confirmation of previous studies, AXDND1 is required for normal manchette structure and thus sperm head shaping [23]. Taken together these data point to a direct role for AXDND1 in generating the tension forces required to shape the sperm nucleus. Of note, the manchette provides dual functions in head shaping and as a scaffold for intra-manchette protein and vesicle transport [4]. As proposed above, data presented here support a role for AXDND1 in the processes of head shaping, protein/vesicle transport along the microtubules of the manchette and ultimately into and along the length of the axoneme and ciliary compartment.

Although the mechanisms of mitochondrial loading into the sperm midpiece are poorly understood, it is hypothesised that the manchette also aids as a scaffold to align the mitochondria parallel to the axoneme prior to their loading onto the ODFs [4]. Dynein is localised to the manchette in early spermatids but its localisation and roles at the time of mitochondrial loading is insufficiently explored [51, 52]. The midpiece presentation in sperm from *Axdnd1*^−/−^ males is, however, more severe than that previously identified in knockout models wherein the manchette is dysfunctional [30, 53]. The severe mitochondrial sheath defects in *Axdnd1*^−/−^ males bear resemblance to sperm from models where sperm individualisation is defective [49, 50], raising the possibility it is a secondary effect of failed individualisation. However, given that defects were evident during mitochondrial sheath development in spermatids, and the localisation of AXDND1 to the developing spermatid midpiece, it remains a possibility that AXDND1 plays a direct role in facilitating the loading of mitochondria into the midpiece compartment.

One caveat of this study was the inability to phase the potential compound heterozygous variants in *AXDND1* patients 2 and 3. In support of their potential pathogenicity, however, the clinical presentation of both men was azoospermia, which is a match with patient 1 and the *Axdnd1* knockout mouse. Patient 3 presented with severe hypospermatogenesis and patient 2 with Sertoli cell only histology. Hypospermatogenesis leading to an eventual Sertoli cell only phenotype was observed in the knockout mouse model, highlighting the benefits of studying the evolving phenotype with age.

In addition to providing significant new data and insights into the role of AXDND1 in sperm tail development in this study [24], we identify that AXDND1 is critically required to sustain the balance between spermatogonial maintenance and commitment to spermatogenesis. The loss of AXDND1 resulted in a progressive loss of germ cell content and a breakdown of the blood–testis barrier that led to immune infiltration. Sperm produced during the first wave were immotile due to abnormal axoneme structure, including the presence of ectopic vesicles, loss of a portion of the outer dense fibres and microtubule doublets, abnormal mitochondrial sheath and sperm head formation, and poor sperm individualisation. Our data shed light on previously unappreciated functions of AXDND1 in male germ cell development and suggest that it functions in protein transport as an atypical component of the cytoplasmic dynein complexes. Finally, we identified an additional four potentially compound heterozygous biallelic variants of unknown significance in *AXDND1*, in men suffering non-obstructive azoospermia suggesting that the insights into the function of AXDND1 in the mouse apply to humans.

### Supplementary information

Combined supplementary material PDF

## Data Availability

Data are available upon request to the corresponding author.
