# Supplementary material for: AXDND1 is required to balance spermatogonial commitment and for sperm tail formation in mice and humans
Source: Cell Death Dis. 2024 Jul 12;15(7):499. doi: 10.1038/s41419-024-06874-5 (PMC11245616; doi:10.1038/s41419-024-06874-5)
Supplement: Supplementary file 1 — Combined supplementary material PDF [file 41419_2024_6874_MOESM1_ESM.pdf]

Supplementary information

Supplementary Figure 1. *AXDND1* and *Axdnd1* coding transcripts. As sourced from Ensembl, human (201, 202, 208, 210, 213 and 214) and mouse (202, 204, 206, 207, 210 and 211) transcripts are shown. White boxes denote 5' and 3' untranslated regions, coloured boxes denote exon regions and numbers specify exon number relative to the main transcript – 201 in humans and 211 in mice. Translated protein size is shown on the right in kDa.

50 word text: Human and mouse coding transcripts for *AXDND1* / *Axdnd1* and predicted protein sizes.

Supplementary Figure 2. Additional defects in *Axdnd1*$^{-/-}$ testes. Stage IX tubules in PAS-stained **A.** Wild type and **B.** *Axdnd1*$^{-/-}$ testes. Black arrows denote retained spermatids. **C.** Wild type and **D.** *Axdnd1*$^{-/-}$ testis cryo-sections stained with CD45 antibody (interstitial immune cells; green) and smooth muscle actin (basement membrane; red). **E.** Caspase 3 and 7 positive cells per tubule in wild type and *Axdnd1*$^{-/-}$ testis sections, as a function of age. * = $p < 0.05$ compared to wild type at the same age point. **F.** Seminal vesicle weight at 70 and 180 days of ago. **G.** AXDND1 localisation in elongating spermatids (green), DNA stained with DAPI (blue). HTCA = head-tail coupling apparatus. **H.** Wild type and **I.** *Axdnd1*$^{-/-}$ cauda epididymis cellular content with Giemsa stain. Black traced circles indicate likely immune cells. Scale bars = 20 µm. Data (all n ≥ 3 animals/time point/genotype) are presented as mean ± SD.

50 word text: Loss of AXDND1 cause a retention of elongating spermatids, increased apoptosis and immune cell infiltration into the testis.

Supplementary Figure 3. A second knockout line confirms AXDND1 is essential for male fertility. *Axdnd1* 571 knockout = *Axdnd1*^{del2} line**.** Reproductive parameters were assessed at 10-12 weeks of age. **A.** Litter size. **B.** Body weight. **C.** Testis weight. **D.** Epididymis weight**. E.** Daily sperm production. **** = $p < 0.0001$. Data (all n ≥ 4 animals/genotype) are presented as mean ± SD.

50 word text: A second loss of function *Axdnd1* strain further supported its role in male fertility, highlighted by male infertility, reduced testis weight and sperm numbers in the testis.

Supplementary Figure 4. AXDND1 is required for normal sperm head shape. Sperm head shape as identified by DAPI staining and objective nuclear morphology software analysis. Sperm from **A.** Wild type and **B.** *Axdnd1*$^{-/-}$. **C.** The proportion of sperm heads classified as cluster 1 (normal), 2 (slightly abnormal) or 3 (abnormal). * = $p < 0.05$, ** = $p < 0.01$, ns = not significant. Data (all n ≥ 3 animals/genotype) are presented as mean ± SD.

50 word text: AXDND1 is essential for sperm head shaping via direct role in manchette function.

Supplementary Figure 5. Novel variants in *AXDND1* in men with azoospermia. Variants of unknown significance are shown in gold, while the likely pathogenic stop-gain variant is shown in red. Periodic acid-Schiff's-stained biopsies from patients M1557 and M2628 are shown, exhibiting a loss of germ cells (Sertoli cell-only) and hypospermatogenesis, respectively. ES = elongating spermatids.

50 word text: Variants identified in the *AXDND1* gene annotated with the coding regions of the transcript / protein and histology of the infertile men.

Supplementary Table 1. Primer sets used in this study.

**Primer set**	**Forward**	**Reverse**
Axdnd1 tissue series qPCR	CTCGGTCACCAGCAATGAAA	CAGACAGTGGTGAGGCCAAG
Ppia qPCR	GTCTCCTTCGAGCTGTTT	ACCCTGGACATGAATCCT
Genotyping primers<br>*Wild type band*<br>*Knockout band*	 GGCAACCTTTACTGGAAGCCTG<br>CCCCGCCCAGAGAGTTTGTA	 CCTCGTTTCCCCTGCGTATCT<br>ACTCTGAATTTTCCATGAGAAGGGA
Guide RNAs	**Upstream of exon 5**	**Downstream of exon 5**
	TGCCATTTAAGGACACGTA	GACTGTAACAGCAGCCCGCA
**Primer set**	**Forward**	**Reverse**
Axdnd1$^{-/-}$ qPCR (long transcript 211)	CAGCACATGTTCACACCTCA	GGAGGTTTTTCTGGGTCAAA
Axdnd1$^{-/-}$ qPCR (short transcript 208)	CCTGGGTTCCCGCCTATTAC	CATCTGCTGGGCGATATGGT

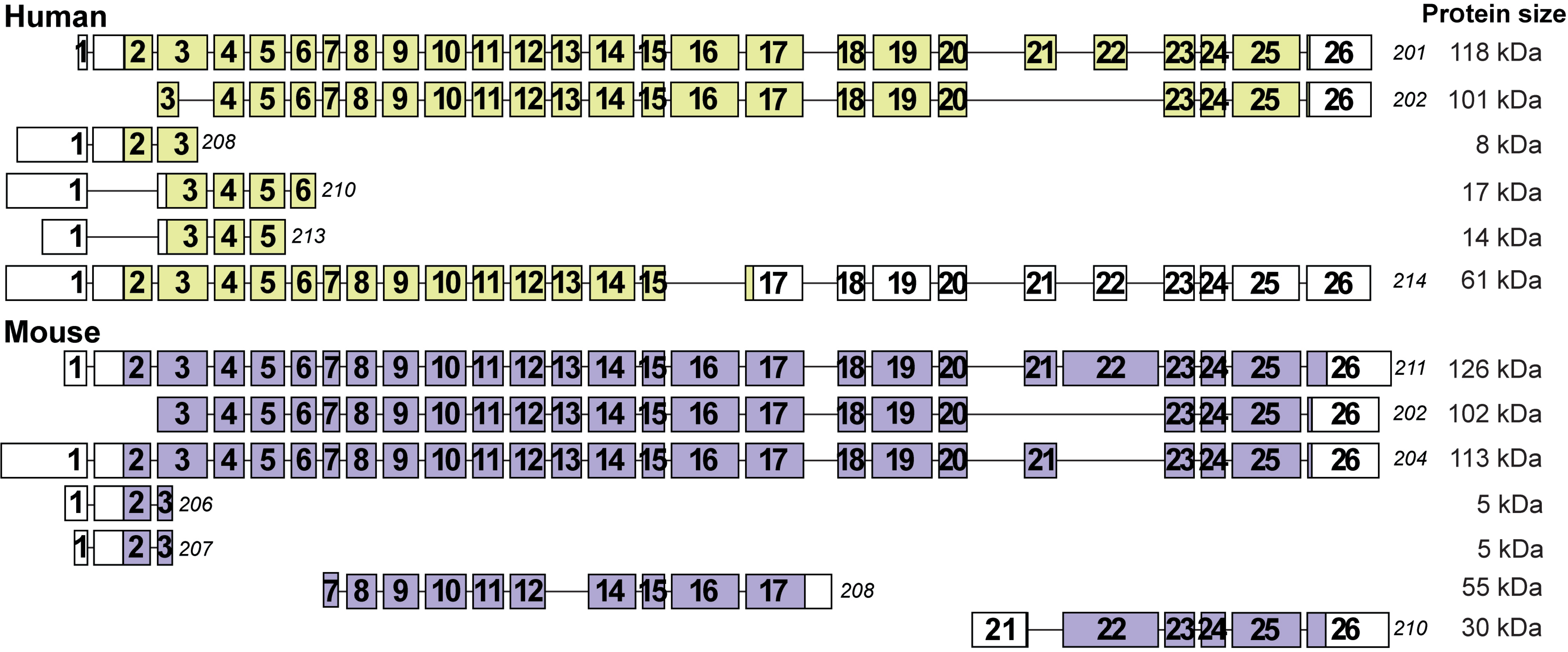
Human
Protein size
201 118 kDa
202 101 kDa
208 8 kDa
210 17 kDa
213 14 kDa
214 61 kDa
Mouse
211 126 kDa
202 102 kDa
204 113 kDa
206 5 kDa
207 5 kDa
208 55 kDa
210 30 kDa

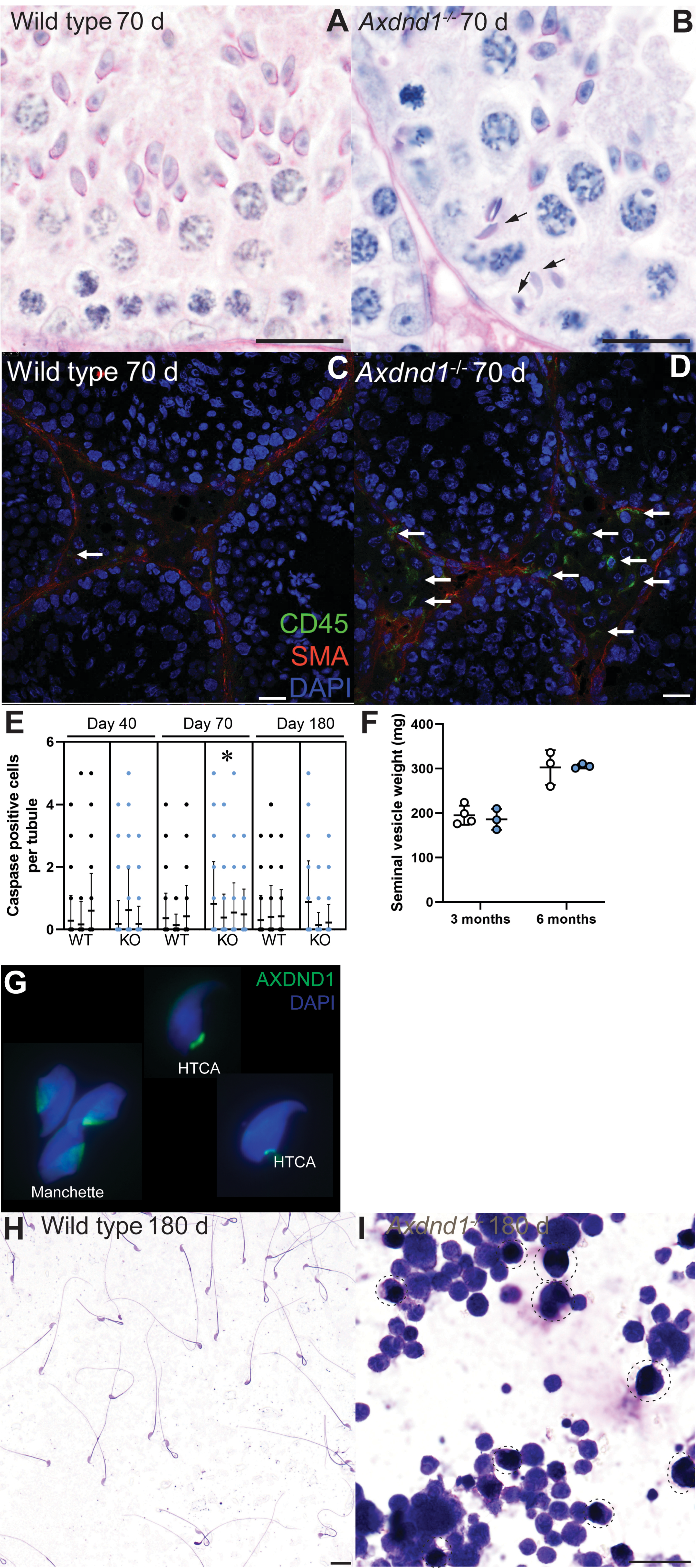

Wild type 70 d
A
Axdnd1-/- 70 d
B
Wild type 70 d
C
Axdnd1-/- 70 d
D
CD45
SMA
DAPI
E
Day 40
Day 70
Day 180
Caspase positive cells per tubule
WT
KO
WT
KO
WT
KO
F
Seminal vesicle weight (mg)
3 months
6 months
G
AXDND1
DAPI
HTCA
Manchette
HTCA
H
Wild type 180 d
I
Axdnd1-/- 180 d

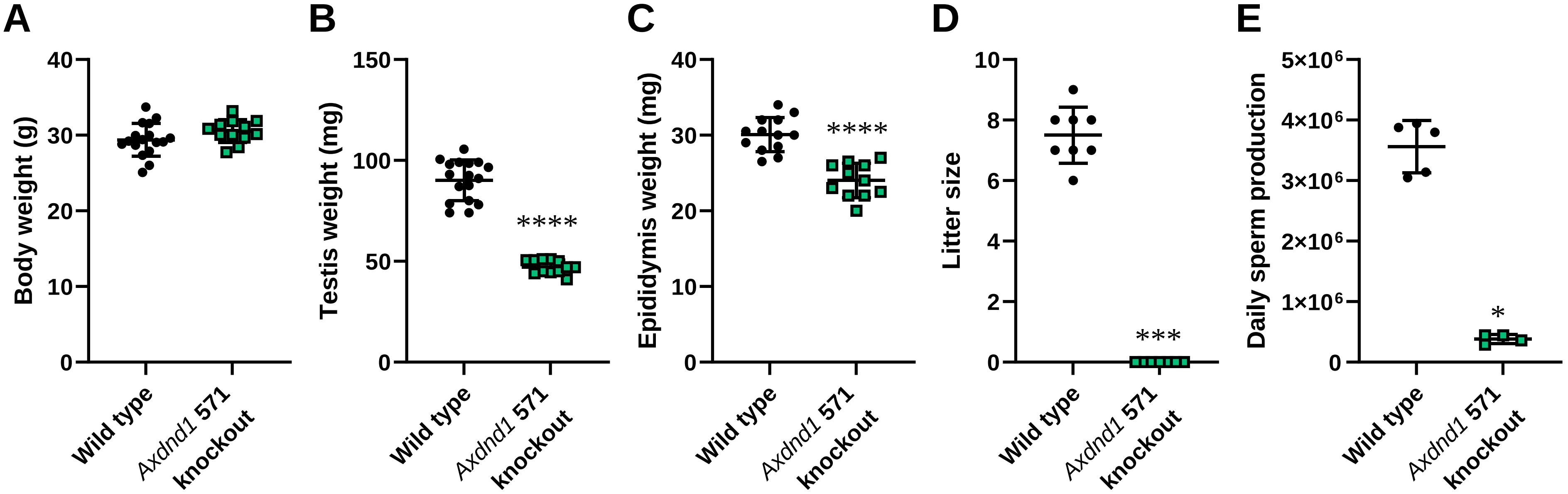
A
B
C
D
E
Body weight (g)
Testis weight (mg)
Epididymis weight (mg)
Litter size
Daily sperm production
****
****
***
*
Wild type
Axdnd1 571 knockout

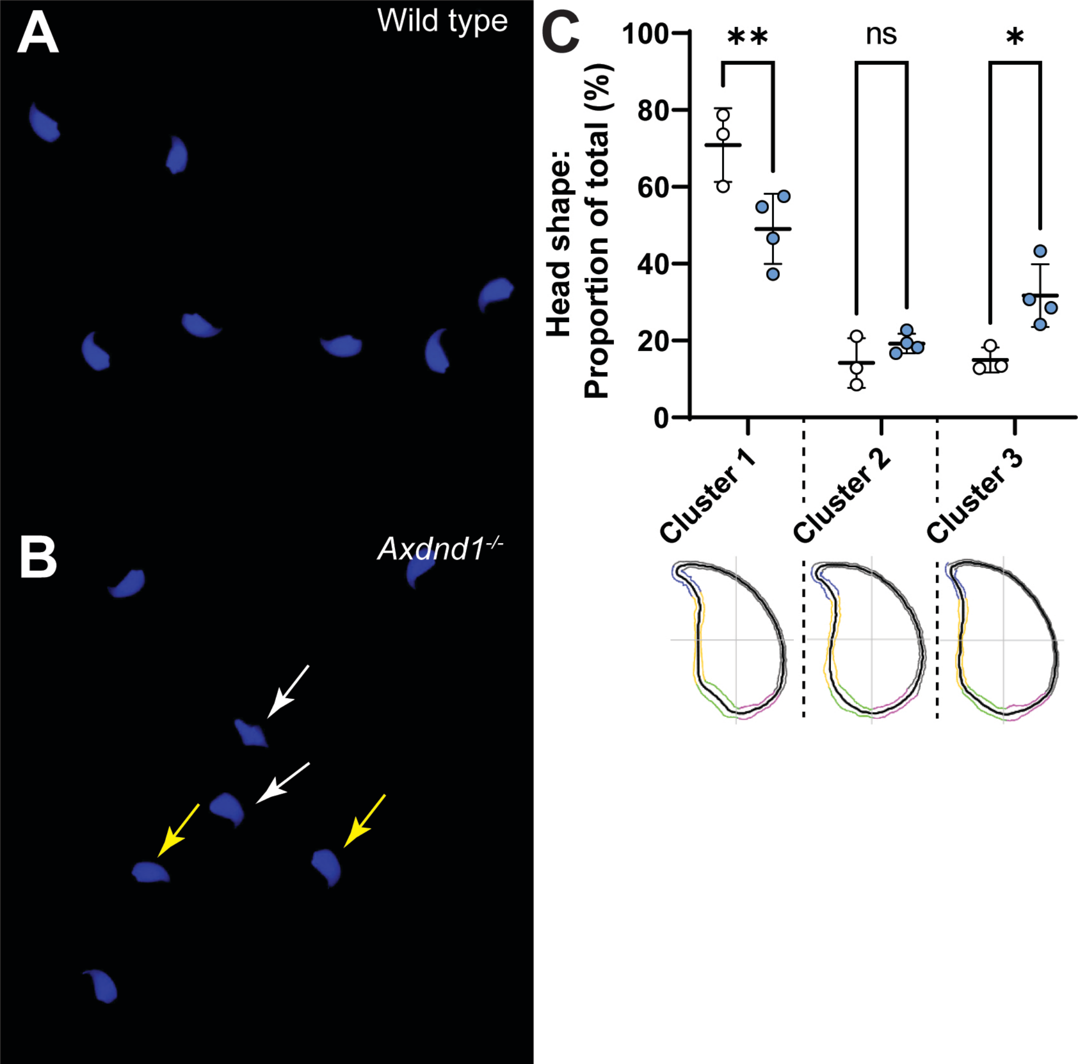
A
Wild type
B
Axdnd1-/-
C
Head shape: Proportion of total (%)
100
80
60
40
20
0
**
ns
*
Cluster 1
Cluster 2
Cluster 3

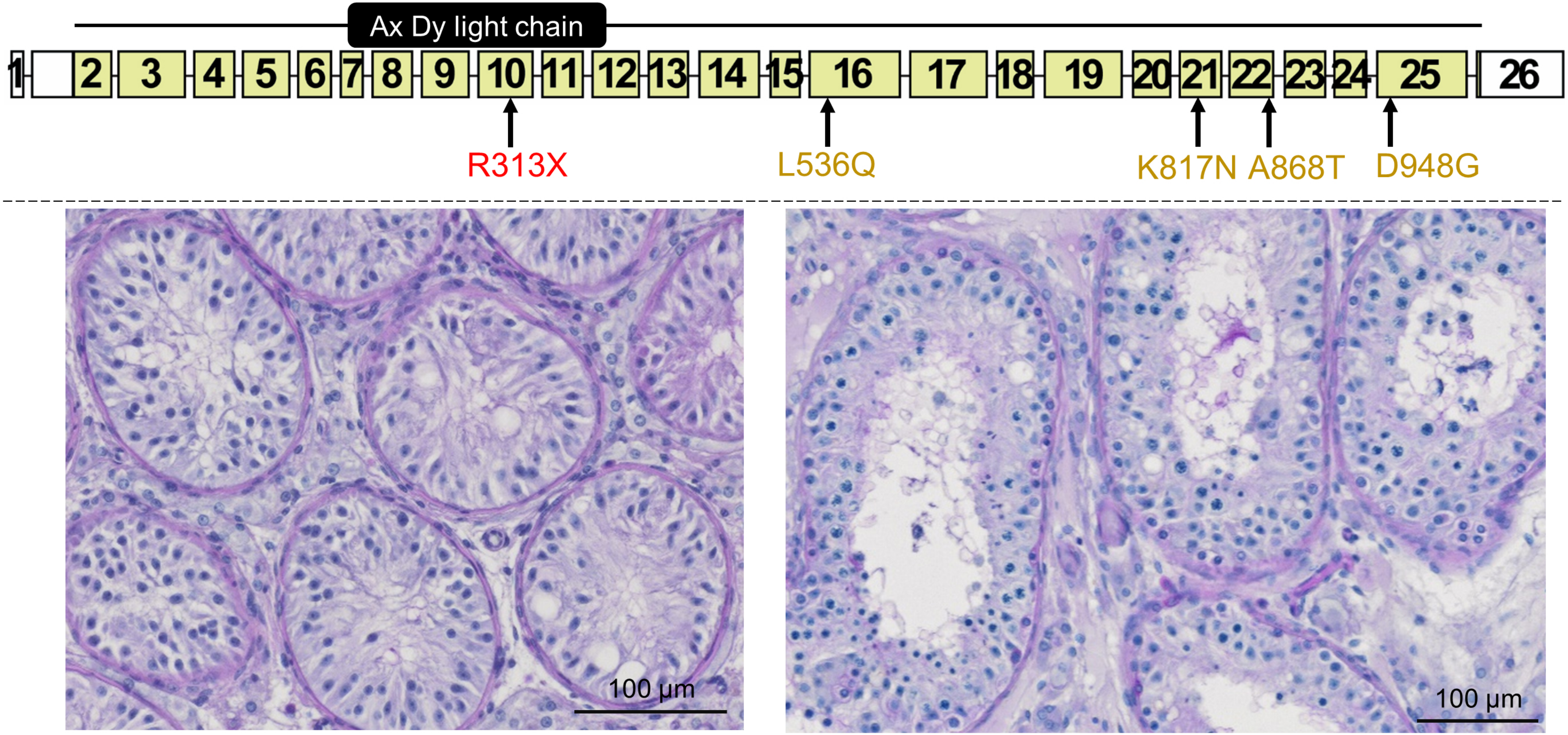

Patient 2 (M1557). Sertoli cell only

Patient 3 (M2628). 50% of tubules with ES
